# Quantification of CD4^+^ T Cell Alloreactivity and Its Control by Regulatory T Cells Using Time‐Lapse Microscopy and Immune Synapse Detection

**DOI:** 10.1111/ajt.13607

**Published:** 2016-01-29

**Authors:** S. C. Juvet, S. Sanderson, J. Hester, K. J. Wood, A. Bushell

**Affiliations:** ^1^ Transplantation Research Immunology GroupNuffield Department of Surgical SciencesJohn Radcliffe HospitalUniversity of OxfordOxfordUK; ^2^ Toronto Lung Transplant Program and Division of RespirologyDepartment of MedicineUniversity Health Network and University of TorontoTorontoOntarioCanada; ^3^ NIHR BRC Translational Immunology LaboratoryNuffield Department of MedicineJohn Radcliffe HospitalUniversity of OxfordOxfordUK; ^4^ Present address: Toronto General HospitalTorontoOntarioCanada

## Abstract

Assays designed to select transplant recipients for immunosuppression withdrawal have met with limited success, perhaps because they measure events downstream of T cell–alloantigen interactions. Using *in vitro* time‐lapse microscopy in a mouse transplant model, we investigated whether transplant outcome would result in changes in the proportion of CD4^+^ T cells forming prolonged interactions with donor dendritic cells. By blocking CD4–MHC class II and CD28–B7 interactions, we defined immunologically relevant interactions as those ≥500 s. Using this threshold, T cell–dendritic cell (T‐DC) interactions were examined in rejection, tolerance and T cell control mediated by regulatory T cells. The frequency of T‐DC contacts ≥500 s increased with T cells from mice during acute rejection and decreased with T cells from mice rendered unresponsive to alloantigen. Regulatory T cells reduced prolonged T‐DC contacts. Importantly, this effect was replicated with human polyclonally expanded naturally occurring regulatory T cells, which we have previously shown can control rejection of human tissues in humanized mouse models. Finally, in a proof‐of‐concept translational context, we were able to visualize differential allogeneic immune synapse formation in polyclonal CD4^+^ T cells using high‐throughput imaging flow cytometry.

## Abstract 2

The authors measure mouse and human CD4+ T cell alloreactivity using time‐lapse microscopy and imaging flow cytometry techniques, and demonstrate that these measurements predict graft outcome in tolerized and rejecting animals. See the supporting video at amjtransplant.com/videos.

**Abbreviations**
APC: antigen‐presenting cell
BMDC: bone marrow–derived dendritic cell
CFP: cyan fluorescent protein
DC: dendritic cell
DST: donor‐specific transfusion
FITC: fluorescein isothiocyanate
GFP: green fluorescent protein
GM‐CSF: granulocyte macrophage colony‐stimulating factor
MoDC: monocyte‐derived dendritic cell
PBMC: peripheral blood mononuclear cell
rh: recombinant human
T‐DC: T cell–dendritic cell
Teff: effector T cell
Treg: regulatory T cell

## Introduction

Marked patient‐to‐patient differences exist in the immunosuppression required to prevent allograft rejection 1, 2. Many assays have been developed in an attempt to predict rejection or to identify operationally tolerant patients 3. The mixed leukocyte reaction, which measures recipient T cell proliferation in response to donor antigens, is poorly predictive 4, 5, although deep sequencing of recipient TCRs in pretransplant mixed leukocyte reactions was recently found to be predictive of tolerance in a small group of patients 6. Limiting dilution assays, cytokine enzyme‐linked immunospot assays and the transvivo assay are either impractical or measure a narrow range of phenomena that may inadequately reflect donor reactivity 7, 8, 9, 10, 11, 12. Transcriptomics methods have shown promise in several cohorts 13, 14, 15, 16, 17, 18, but important differences across studies 19 raise questions about the practicality of this approach. Better tools to assess donor reactivity in individual patients are urgently needed to allow informed decisions about immunosuppression minimization.

In many transplant models, sustained allograft survival depends on regulatory T cells (Tregs) 20, 21, 22, 23, and immunosuppression weaning in some patients might involve such populations. Tregs control autoimmunity by inhibiting stable immune synapse formation between T cells and dendritic cells (DCs) 24, partly because autoreactive Tregs make prolonged contacts with DCs 25, depriving effector T cells (Teffs) of the sustained contacts required for activation 24, 26. Whether these phenomena characterize alloreactive T cell and Treg behavior has not been studied systematically but is important because Treg cellular therapy is currently the subject of a phase I/IIa clinical trial in renal transplant recipients 27.

Using *in vitro* time‐lapse microscopy to examine mouse T cell–DC (T‐DC) interactions, we tested the hypothesis that the frequency of prolonged contacts between recipient CD4^+^ T cells and donor DCs *in vitro* reflects allograft rejection and tolerance. Using antibodies or costimulatory blockade, we demonstrated that a threshold of 500 s distinguishes brief sampling interactions from those that drive a productive T cell response. Allograft rejection and tolerance were associated with predictable changes in the proportion of interaction events ≥500 s. Furthermore, human CD4^+^ T cells exhibited similar behavior in response to allogeneic DCs. Finally, we showed that imaging flow cytometry can be used to examine CD4^+^ T cell alloreactivity, suggesting that the state of T cell responsiveness to donor antigens could be evaluated in a high‐throughput manner. Our observations imply that measuring the frequency of prolonged T‐DC contact or immune synapse formation *in vitro* might be a useful measure of donor reactivity in transplant recipients.

## Materials and Methods

### Mice

CBA/Ca (CBA, H‐2^k^), CBA Foxp3‐GFP, CBA RAG^−/−^, C57BL/6 (B6, H‐2^b^) B6.129(ICR)‐Tg(CAG‐ECFP)CK6Nagy/J (B6 CFP) mice were bred in the John Radcliffe Hospital Biomedical Services unit. This study was conducted under Home Office project license 40/3580.

### Reagents and mAbs

Fluorochrome‐conjugated mAbs were from eBioscience (Hatfield, UK). Anti‐mouse CD4 (YTS177) and anti‐mouse CD25 (PC61) mAbs were produced in‐house. Anti‐human CD4 mAb TRX1 was a gift from Prof. S. Cobbold, University of Oxford, Oxford, UK. Abatacept (CTLA4‐Ig; Orencia) was from Bristol Myers Squibb (Uxbridge, UK). Recombinant mouse granulocyte macrophage colony‐stimulating factor (GM‐CSF), human GM‐CSF, human TGF‐β1 and human IL‐4 were from Peprotech EC (London, UK).

### Tolerance induction and transplantation

CBA Foxp3‐GFP mice were untreated or received YTS177 200 μg intravenously on days −28 and −27 and/or 200 μL B6 heparinized whole blood as a donor‐specific transfusion (DST) on day −27 and sometimes also on day −1. Some mice received PC61 1 g intraperitoneally on day −14. At day 0, mice received a heterotopic B6 cardiac allograft, as described 28, or were euthanized to obtain CD4^+^ T cells. Cessation of cardiac pulsation signified rejection (approximately day 8 in untreated animals). For skin grafting, B6 tail skin grafts were applied to the left flank of CBA or CBA RAG^−/−^ mice. Dressings were removed at day 6, and grafts were monitored two or three times weekly for rejection.

### Cell sorting

Mouse CD4^+^ T cells were sorted to ≥98% purity on a FACSAria (Becton Dickinson, Oxford, UK). Human CD4^+^ T cells were negatively selected to >90% purity from peripheral blood mononuclear cells (PBMCs) by removing CD8^+^, CD25^+^, CD56^+^, CD14^+^, and CD19^+^ populations (FACSAria; Becton Dickinson).

### Human PBMCs and expanded Tregs

Buffy coats (UK National Blood Service, Watford, UK) were separated on Ficoll‐Paque (GE Healthcare, Little Chalfont, UK). PBMCs were cryopreserved prior to recovery and sorting. Separately, autologous CD4^+^CD25^hi^CD127^lo^ Tregs were sorted and expanded prior to cryopreservation, as described 29. Recovered Tregs were labeled with 1 μM CMPTX (Life Technologies, Paisley UK). All blood donors provided informed consent (UK National Research Ethics Service, Oxford, UK).

### DCs

Immature mouse bone marrow–derived DCs (BMDCs) were generated with recombinant mouse GM‐CSF (2 ng/mL) and recombinant human (rh) TGF‐β (2 ng/mL) 30. Immature human monocyte‐derived DCs (MoDCs) were generated from healthy volunteer CD14^+^ monocytes with rhGM‐CSF (50 ng/mL) and rhIL‐4 (1000 U/mL) 31. All DCs were cryopreserved and recovered on the day of the experiment.

### Time‐lapse microscopy and image analysis

Time‐lapse microscopy was performed as described by Sarris et al 32 with minor modifications. T cells (total 2 × 10^5^, in some cases 1 × 10^5^ conventional T cells and 1 × 10^5^ Tregs) were added to 1 × 10^5^ (5 × 10^4^ for human MoDCs) allogeneic DCs in 200 μL indicator‐free RPMI containing 20 mM HEPES (Sigma‐Aldrich, St. Louis, MO) in eight‐well chambers (Lab‐Tek II; Nalge Nunc International, Roskilde, Denmark). Imaging was performed on a Deltavision Elite Imaging System (Applied Precision; Imaging Solutions, Preston, UK). Polarized light, green fluorescent protein (GFP) and cyan fluorescent protein (CFP) images (×20) were acquired sequentially every 20 s for 40 min (120 frames). The number of contiguous frames in which individual T cells and DCs were in contact was enumerated and expressed as contact duration (in seconds). For each recording, all T cells contacting 16 individual DCs were analyzed, typically providing ≥100 T‐DC interaction events. T cells that appeared to be caught passively in DC clusters were excluded from the analysis.

### Detection of immune synapse formation using imaging cytometry

CBA splenic CD4^+^ T cells were obtained by negative selection (≥85% purity). CBA and B6 BMDCs were cocultured (2:1 ratio) for 4 h at 37°C. Cells were fixed with 1% formaldehyde, stained with anti‐CD90.2 allophycocyanin and anti‐CD11b eFluor 450, permeablized with Triton X‐100 in phosphate‐buffered saline and stained with 7AAD and phalloidin and fluorescein isothiocyanate (FITC; 0.05 μg/mL) prior to acquisition on an ImageStream IS100 (Amnis Corporation, San Diego, CA). Data were analyzed using IDEAS software (Amnis Corporation), as described in Figure S1.

### Statistical analysis

Statistical analysis was performed using GraphPad Prism 5 (GraphPad Software, San Diego, CA). Proportions of T‐DC interaction times ≥500 s were compared by Fisher exact test or χ^2^ test. Graft survival data were analyzed using the log‐rank test. Means of two groups were compared using paired or unpaired t‐tests according to the experimental design.

## Results

### A threshold of 500 s discriminates immunologically relevant T‐DC interactions in a polyclonal T cell population

**Figure 1:**
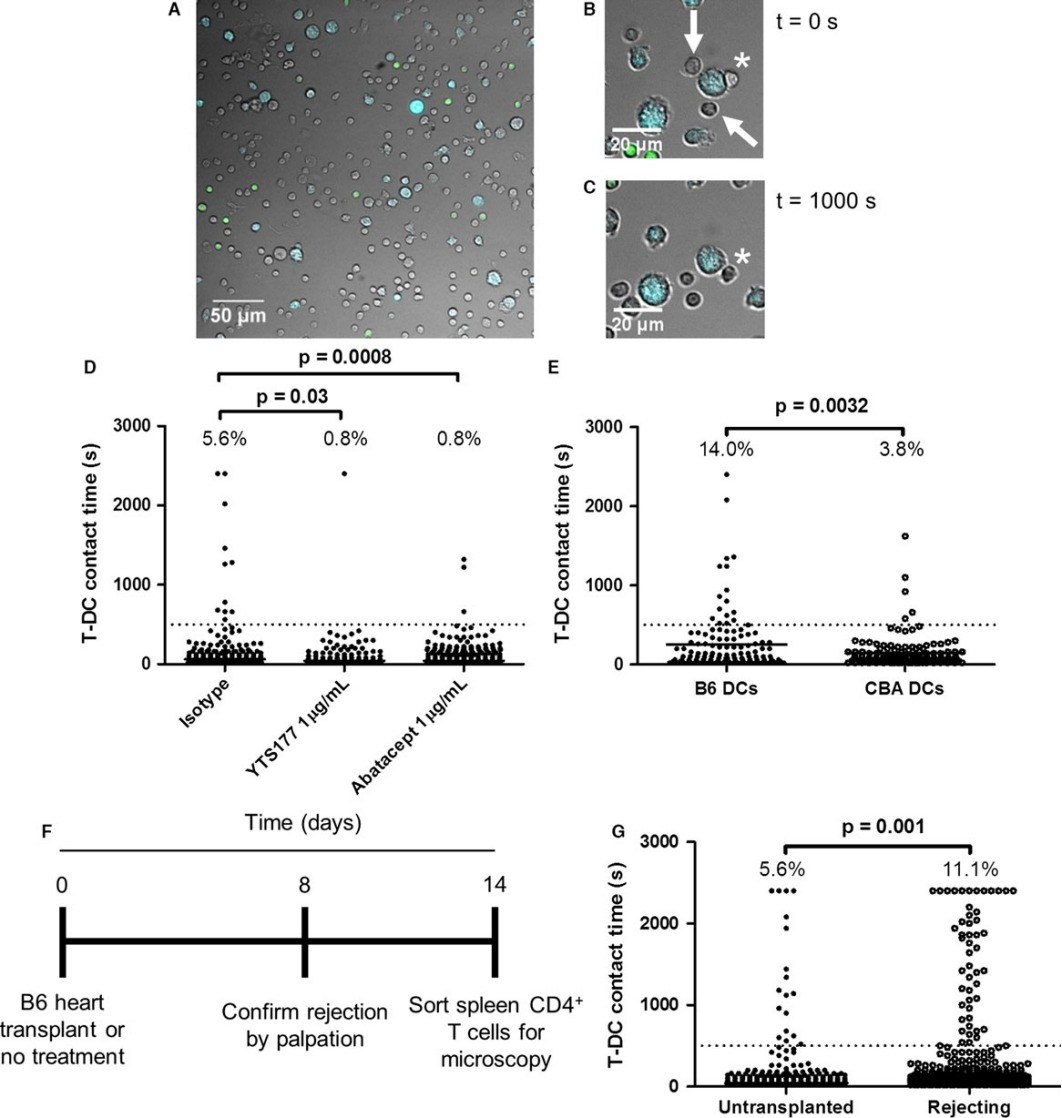
**A 500‐s threshold discriminates synapse‐dependent interactions from synapse‐independent ones.** CBA Foxp3‐GFP T cells were combined with B6 CFP DCs (2 × 10^5^ to 1 × 10^5^) in 200 μL media in a microscopy chamber and imaged at ×20 magnification every 20 s for 40 min at 37°C. (A) First image in a representative recording shows effector T cells (colorless), regulatory T cells (green) and DCs (blue). Original magnification ×20. (B) Enlarged images show three T cells interacting with a DC in the first frame (t = 0 s). (C) Two of these (white arrows) interacted only briefly and are not present at t = 1000 s; the T cell marked by the asterisk maintains contact with the DC during this time. (D) Distribution of T‐DC contact times in the presence of YTS177 at 1 μg/mL (middle column), its isotype control (left column) or abatacept at 1 μg/mL. Dotted line at t = 500 s indicates the threshold above which contacts were nearly completely eliminated by YTS177 or abatacept. Numbers at the top of each column are the percentage of events >500‐s threshold. Fisher exact test, p = 0.03 (isotype control vs. YTS177) and p = 0.0008 (isotype control vs. abatacept). Data are from one of three independent experiments with similar results. (E) CBA Foxp3‐GFP T cells were combined with B6 CFP DCs and CBA (CFP ^−^) DCs (2 × 10^5^ to 5 × 10^4^:5 × 10^4^) in 200 μL media in a microscopy chamber and imaged at ×20 magnification every 20 s for 40 min at 37°C. T‐DC contact times with B6 and CBA DCs are shown. Fisher exact test, p = 0.0032. Data are from one of three independent experiments with similar results. (F and G) CBA mice uniformly rejected B6 cardiac allografts by day 8; other CBA mice were left untreated. At day 14, contact times between their splenic T cells and B6 CFP DCs were measured. (F) Experimental timeline. (G) Compiled interaction times for untransplanted (n = 2) and transplanted (n = 3) mice in 2 independent experiments. Percentage of events ≥500 s is shown above each column. Fisher exact test, p = 0.001. CFP, cyan fluorescent protein; DC, dendritic cell; T‐DC, T cell–dendritic cell.

To measure allogeneic T‐DC contact duration, we sorted CD4^+^ T cells from CBA Foxp3‐GFP mice, combined them with B6 CFP BMDCs and recorded serial ×20 images every 20 s over 40 min. This allowed us to distinguish Foxp3^−^ T cells (colorless), Foxp3^+^ Tregs (green) and DCs (blue) (Figure 1A and Video S1). Although most effector T‐DC interactions were short‐lived, contacts lasting the entire length of the recording were also seen (Figures 1B–D).

T cell activation requires prolonged TCR signaling 33, 34, triggering a “stop” signal that arrests T cells on antigen‐presenting cells (APCs) 35. We reasoned that blockade of TCR–MHC or costimulatory interactions could be used to define a minimum contact duration required for T cell activation. Consequently, we assessed the effect of a CD4 antibody (YTS177) that blocks CD4^+^ T cell activation by alloantigen 36 and CTLA4‐Ig (abatacept), which blocks CD28 costimulation 37, on T‐DC contact durations. Both reagents markedly reduced long T‐DC interactions (Figure 1D) but had no discernable effect on interactions <500 s (dotted line in Figure 1D). Based on this observation and similar findings with MHC class II^−/−^ DCs 25, we defined a threshold of 500 s to discriminate immunologically irrelevant, short‐lived contacts from prolonged interactions likely to result in T cell activation.

We reasoned that T‐DC contact times would differ substantially if T cells were given the opportunity to interact with either allogeneic or syngeneic DCs. To assess this possibility, we combined CBA T cells with a 1:1 mixture of B6 (CFP^+^) and CBA (CFP^−^) DCs. As expected, there were proportionately more prolonged allogeneic T‐DC contacts than syngeneic ones (Figure 1E), indicating that this method is capable of distinguishing alloreactivity from self‐reactivity.

Graft rejection depends on alloreactive T cell expansion. We predicted that CD4^+^ T cells from animals that had rejected an allograft would exhibit an increased proportion of prolonged T‐DC interactions. Untreated CBA Foxp3‐GFP mice were untransplanted or given B6 cardiac allografts. Rejection was confirmed by palpation on day 8. To allow acute inflammation to subside, CD4^+^ T‐DC interaction times were measured on day 14 (timeline in Figure 1F). Rejecting animals exhibited a twofold increase in the proportion of long interactions, from 5.6% to 11.1% (Figure 1G and Video S2). Consequently, an increased proportion of prolonged T‐DC contacts detected *in vitro* correlates with allograft rejection *in vivo*.

### An increase in long T‐DC interactions precedes the onset of visible allograft rejection

Allograft biopsies are invasive, and because rejection is a diffuse process, multiple biopsies are often required 38. Allograft rejection is often assessed using clinical indices of organ function 38, but these usually reflect rather than predict organ damage. Detection of increased alloreactivity before organ dysfunction develops could provide an enhanced opportunity for therapeutic intervention.

**Figure 2:**
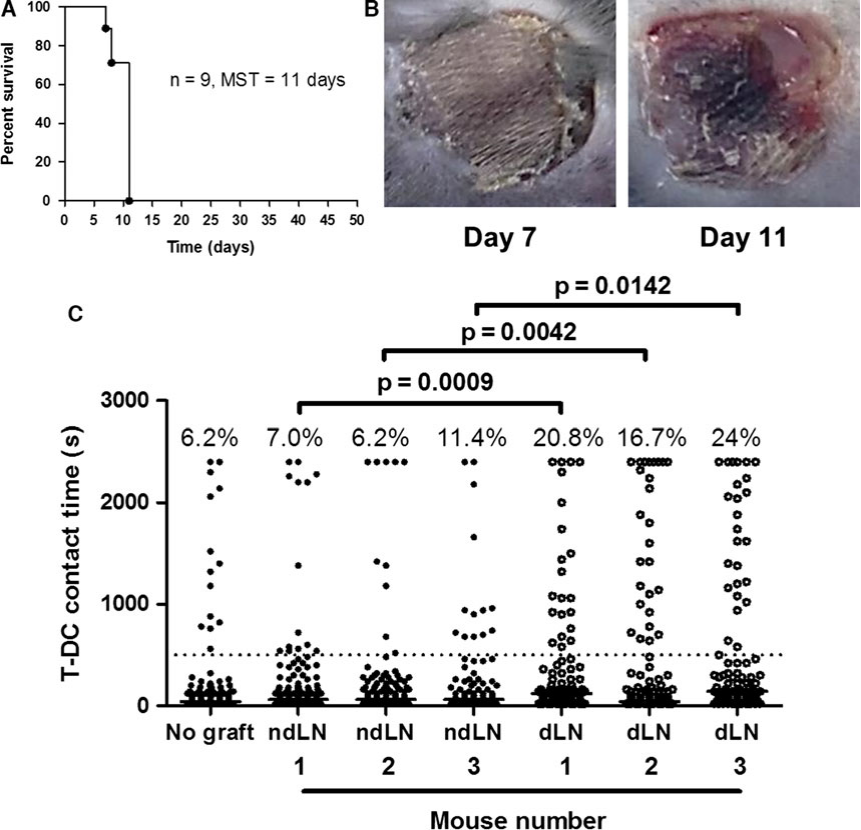
**An increase in long T‐** **DC** **interactions precedes the onset of visible allograft rejection.** Nine CBA mice were given B6 skin grafts. (A) Graft survival is shown. Median survival time (MST) was 11 days. (B) Eight of the nine mice had no visible skin allograft rejection at day 7 (left panel), and three of these were euthanized for analysis; the remaining animals uniformly rejected their grafts by day 11 (right panel). (C) Graft‐ipsilateral dLN and contralateral ndLN CD4^+^ T cell interaction times with B6 cyan fluorescent protein DCs were measured at day 7 in three mice with no visible signs of rejection. Interaction times of lymph node CD4^+^ T cells from a fourth mouse (no graft) are shown in the first column for comparison. Percentage of events ≥500 s is shown above each column. For the comparison of dLN to ndLN T cell interaction times with the Fisher exact test, p = 0.0009 (mouse 1), p = 0.0042 (mouse 2), and p = 0.0142 (mouse 3). DC, dendritic cell; dLN, draining lymph node; ndLN, nondraining lymph node; T‐DC, T cell–dendritic cell.

To test the hypothesis that an increase in long T‐DC interactions might predict rejection, naïve CBA mice were given B6 skin grafts. Median graft survival time was 11 days (Figures 2A and B). On day 7, before visible signs of rejection (Figure 2B, left panel), T‐DC interaction times were measured using CD4^+^ T cells from the draining and nondraining axillary lymph nodes of three mice. In each animal, there was a two‐ to threefold (mean 2.6‐fold) increase in prolonged interactions made by draining versus nondraining lymph node CD4^+^ T cells (Figure 2C). Moreover, the proportion of prolonged contacts made by nondraining lymph node CD4^+^ T cells was similar to that of CD4^+^ T cells from a nontransplanted mouse (Figure 2C, first column). In this model, an increase in prolonged T‐DC interactions *in vitro* preceded allograft rejection.

### Tolerance induction results in a decrease in the proportion of stable T‐DC contacts

**Figure 3:**
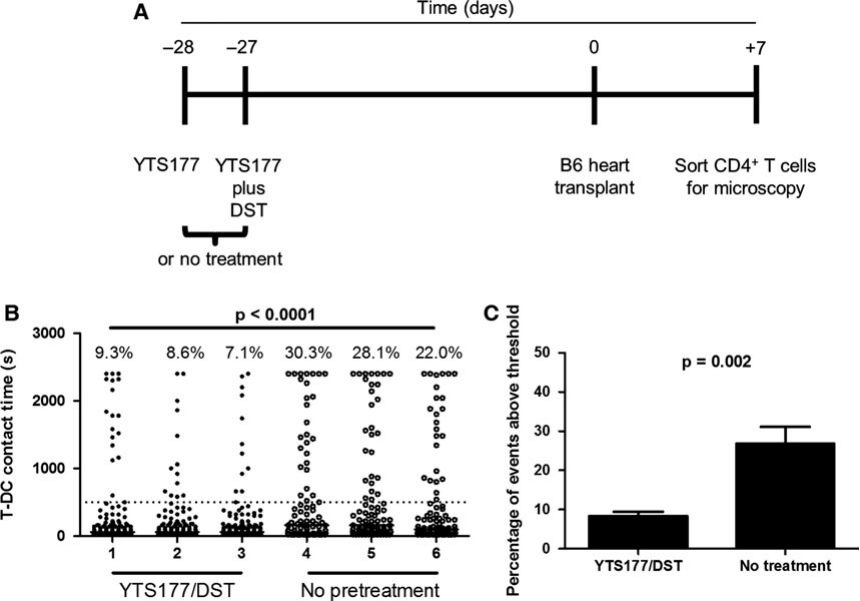
**Induction of allograft tolerance results in a decrease in the proportion of long interactions.** CBA mice were left untreated (n = 3) or rendered unresponsive to B6 alloantigens by administration of 200 μg YTS177 intravenously at days −28 and −27 and 200 μL B6 DST at day −27 (n = 3). All animals then received a B6 cardiac allograft at day 0. On day +7 splenic CD4^+^ T cells were sorted for measurement of interaction time with B6 cyan fluorescent protein dendritic cells. (A) Experimental timeline. (B) Splenic T‐DC interaction times for each mouse are shown. Percentage of events ≥500 s is shown above each column. χ^2^ test, p < 0.0001. (C) The percentage of events ≥500 s for all three mice in each treatment condition is depicted. Student's t‐test, p = 0.002. DST, donor‐specific transfusion; T‐DC, T cell–dendritic cell.

Identification of operationally tolerant patients remains challenging; therefore, we asked whether changes in the proportion of prolonged T‐DC interactions might reflect donor unresponsiveness. CBA mice were rendered unresponsive to B6 cardiac allografts with a nondepleting anti‐CD4 antibody (YTS177) and DST (Figure 3A). Such animals exhibit long‐term allograft survival without further therapy 39. Controls received a B6 cardiac allograft with no pretreatment. Seven days later, recipient splenic CD4^+^ T‐DC interaction times were measured. Figures 3(B) and (C) show that whereas CD4^+^ T cells from nonpretreated mice exhibited many prolonged T‐DC interactions (mean 26.8%), cells from YTS177/DST‐treated mice exhibited a proportion of prolonged T‐DC interactions comparable to those of naïve mice (mean 8.3%; χ^2^ test, p < 0.0001, and t‐test, p = 0.002). Consequently, demonstrable tolerance to alloantigens is associated with a reduction in prolonged T‐DC contacts.

### Decreased T‐DC interactions in animals with induced tolerance are dependent on Tregs

**Figure 4:**
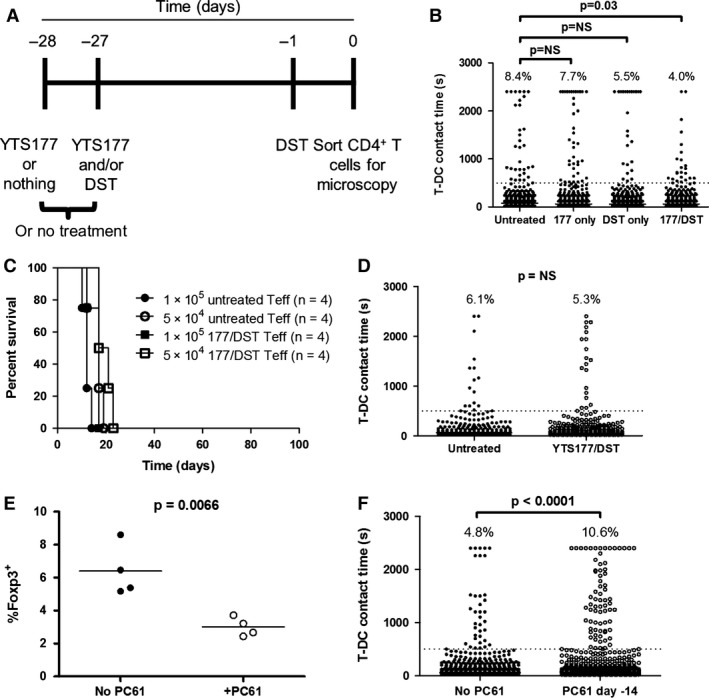
**A tolerance induction protocol decreases T‐** **DC** **interaction time in a regulatory T cell–dependent fashion.** CBA Foxp3‐GFP mice were left untreated, given 200 μg YTS177 on days −28 and −27, given 200 μL B6 DST on day −27, or both. At day −1, all mice (except those that were untreated) received another 200‐μL DST. On day 0, interaction times were measured. (A) Experimental timeline. (B) Compiled interaction times from three independent experiments are shown (CD4^+^ T cells pooled and sorted from two or three mice in each group per experiment). Percentage of events ≥500 s is shown above each column. Fisher exact test, p = 0.03 for the comparison of no treatment and YTS177/DST. For the comparison of no treatment and YTS177 only or DST only, p = NS. (C) 1 or 5 × 10^5^ sorted CD4^+^ GFP ^−^ T cells from untreated or YTS177/DST‐treated mice in panel (A) were injected intravenously into CBA RAG ^−/−^ mice (n = 4 mice per group). The following day, all mice received B6 skin grafts. Graft survival time is shown. Log‐rank test, p = NS. (D) Contact times for CD4^+^ GFP ^−^ T cells with B6 CFP DCs are shown. Percentage of events ≥500 s is shown above each column. Data are compiled from two independent experiments. Fisher exact test, p = NS. (E) CBA Foxp3‐GFP mice were given YTS177/DST, as in panel (A), and then received anti‐CD25 mAb PC61 (1 g intraperitoneally, n = 4) or no treatment (n = 4) on day −14. The percentage of Foxp3‐GFP ^+^ cells within CD4^+^ T cells on day 0 in each group is shown. Data are compiled from two independent experiments. Student's t‐test, p = 0.0066. (F) Interaction times for CD4^+^ T cells from mice in panel (E) with B6 CFP DCs are shown. Percentage of events ≥500 s is shown above each column. Data are compiled from two independent experiments; Fisher exact test, p < 0.0001. CFP, cyan fluorescent protein; DST, donor‐specific transfusion; GFP, green fluorescent protein; NS, not significant; T‐DC, T cell–dendritic cell; Teff, effector T cell.

Treatment of mice with either YTS177 alone or DST alone fails to result in long‐term cardiac allograft survival 39. We predicted that CD4^+^ T cells from mice given only one of these treatments would not exhibit the reduction in prolonged interactions seen with cells from mice given both. To provide an alloantigen challenge, mice received a second DST on day −1 (Figure 4A). T‐DC interactions were measured on day 0. YTS177 alone or DST alone had a modest effect on the proportion of prolonged T‐DC interactions compared with untreated mice. In contrast, tolerance induction reduced the frequency of prolonged interactions by >50% (Figure 4B).

The YTS177/DST protocol generates alloreactive Tregs that arise from the pretreatment alone and are responsible for long‐term allograft survival in both adoptive transfer and primary transplant recipients 20, 22, 40. The effect of YTS177/DST on prolonged T‐DC contacts (Figure 4B) is consistent with regulation by alloreactive Tregs but also could be explained by direct effects on Teffs. To distinguish these possibilities, Foxp3‐GFP mice were given YTS177/DST or left untreated. On day 0, CD4^+^ GFP^−^ T cells were transferred at two doses to CBA RAG^−/−^ mice, which then received B6 skin grafts the following day. Figure 4C shows that untreated and 177/DST GFP^−^ T cells rejected grafts with similar kinetics, arguing against an independent effect of tolerance induction on Teffs. That the reduction in stable contacts shown in Figure 4B is due to Tregs is confirmed by the observation that Treg depletion prior to microscopy completely eliminated the effect of pretreatment on T‐DC interactions (Figure 4D). Consequently, alloantigen‐induced Tregs that regulate allograft rejection *in vivo* also control allogeneic T‐DC interactions *in vitro*.

Treg depletion at day −14 during the YTS177/DST protocol abrogates tolerance 40; therefore, we asked whether this would alter the frequency of prolonged T‐DC interactions on day 0 *in vitro*. CBA Foxp3‐GFP mice received YTS177/DST and were either left untreated or were given anti‐CD25 mAb PC61 on day −14. Importantly, by day −14, alloantigen‐activated Teffs have downregulated CD25 and so are not depleted by PC6l 40. Treg depletion was ≈50% at day 0 (Figure 4E). On day 0, spleen and lymph node total CD4^+^ T cells were sorted, and the interaction times of non‐Treg GFP^−^ T cells with B6 DCs were measured. *In vivo* depletion of Tregs resulted in a 2.2‐fold increase in the proportion of prolonged T‐DC contacts (Figure 4F). Taken together, these data show that *in vitro* time‐lapse microscopy can model tolerance induction *in vivo*.

**Figure 5:**
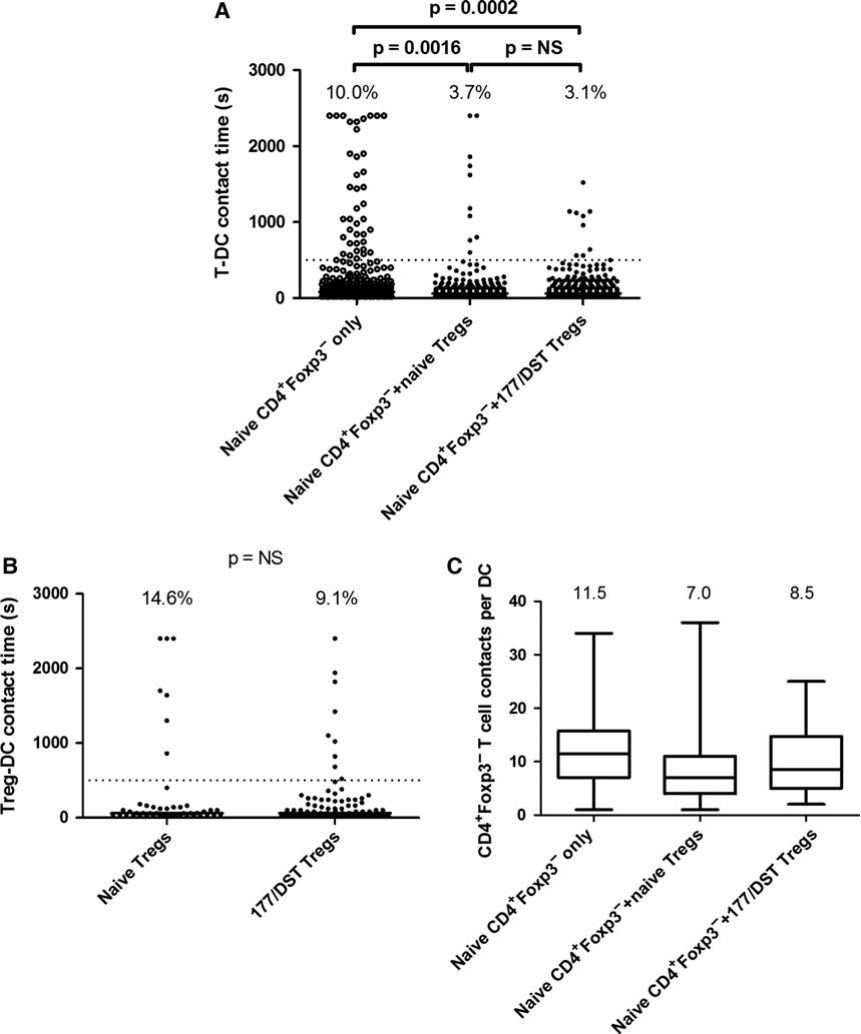
**Tregs control allogeneic T‐** **DC** **interactions** ***in vitro*** **.** CD4^+^ GFP ^−^ Teffs were sorted from untreated CBA mice. CD4^+^ GFP ^+^ Tregs were sorted from untreated and YTS177/DST‐treated mice. (A) Interaction times for GFP ^−^ T cells were measured in the absence of Tregs (2:1 T cell:DC ratio, Teffs only) or in their presence (1:1:1 T cell:Treg:DC ratio) (middle and right columns). Percentage of events ≥500 s is shown above each column. Data are compiled from two independent experiments. Fisher exact test, p = 0.0016 (Teffs only vs. Teffs plus naïve Tregs), p = 0.0002 (Teffs only vs. Teffs plus YTS177/DST Tregs), p = NS (Teffs plus naïve Tregs vs. Teffs plus YTS177/DST Tregs). (B) DC interaction times for Tregs from naïve and YTS177/DST‐treated mice are shown. Data are compiled from two independent experiments (same experiments as in panel A). Fisher exact test, p = NS. (C) Box‐and‐whisker plots illustrating the number of T‐DC contacts per DC in each condition. Top and bottom horizontal bars indicate the maximum and minimum values. Top and bottom of each box represent the 25th and 75th percentiles. The horizontal bar in each box represents the median number of contacts. Numbers above each column are the medians. Data are derived from the two experiments depicted in panel A. DC, dendritic cell; DST, donor‐specific transfusion; GFP, green fluorescent protein; NS, not significant; T‐DC, T cell–dendritic cell; Teff, effector T cell; Treg, regulatory T cell.

We then examined the ability of Tregs from naïve and YTS177/DST‐treated animals to control allogeneic T‐DC contacts *in vitro*. T‐DC interaction times of CD4^+^ GFP^−^ T cells from naïve mice were determined in the presence or absence of Tregs from either naïve or YTS177/DST‐treated mice (1:1 ratio of Tregs:Teffs). The rate of prolonged contact formation for CD4^+^GFP^−^ T cells (10.0%) (Figure 5A, first column) was higher than that seen in Figure 1D, consistent with the fact that Tregs were present in the latter experiment but not in the former. Both Treg populations reduced prolonged T‐DC interactions (Figure 5A, second and third columns). Interactions between Tregs themselves and DCs were not significantly different between the two populations (Figure 5B).

Total T cell numbers were kept constant in these experiments (1 × 10^5^ CD4^+^GFP^−^ and 1 × 10^5^ Tregs vs. 2 × 10^5^ Teffs) to avoid crowding. Conceivably, this could have reduced the opportunity for Teffs to make prolonged contacts with DCs owing to their decreased frequency in the well; however, we observed only a modest (0–30%) reduction in the number of T‐DC contacts made by GFP^−^ T cells in the presence of Tregs (Figure 5C), despite a threefold reduction in the proportion of prolonged contacts (Figure 5A). Despite their decreased density when Tregs were added, GFP^−^ T cells had a similar opportunity to form prolonged DC contacts, confirming a Treg‐dependent inhibition of prolonged contact formation.

In autoimmune models, Tregs can displace conventional T cells from DCs 41. Within cultures containing Tregs, we compared T‐DC interaction times between DCs visited and not visited by Tregs. We observed a nonsignificant decrease in the lengths of prolonged (≥500 s) interactions with DCs visited and not visited by Tregs (median 1330 vs. 884 s, respectively), but there was no difference in the proportion of interactions ≥500 s (both ≈3%) (data not shown).

Taken together, these data are consistent with previous observations 24, 26, 41 and the results in Figure 4. They also concur with our previous work showing that naturally occurring Tregs can be as effective as YTS177/DST‐driven Tregs at preventing skin graft rejection, with potency differences emerging only at high Teff:Treg ratios 40.

### Human T cells exhibit allogeneic T‐DC interactions that can be controlled by Tregs

**Figure 6:**
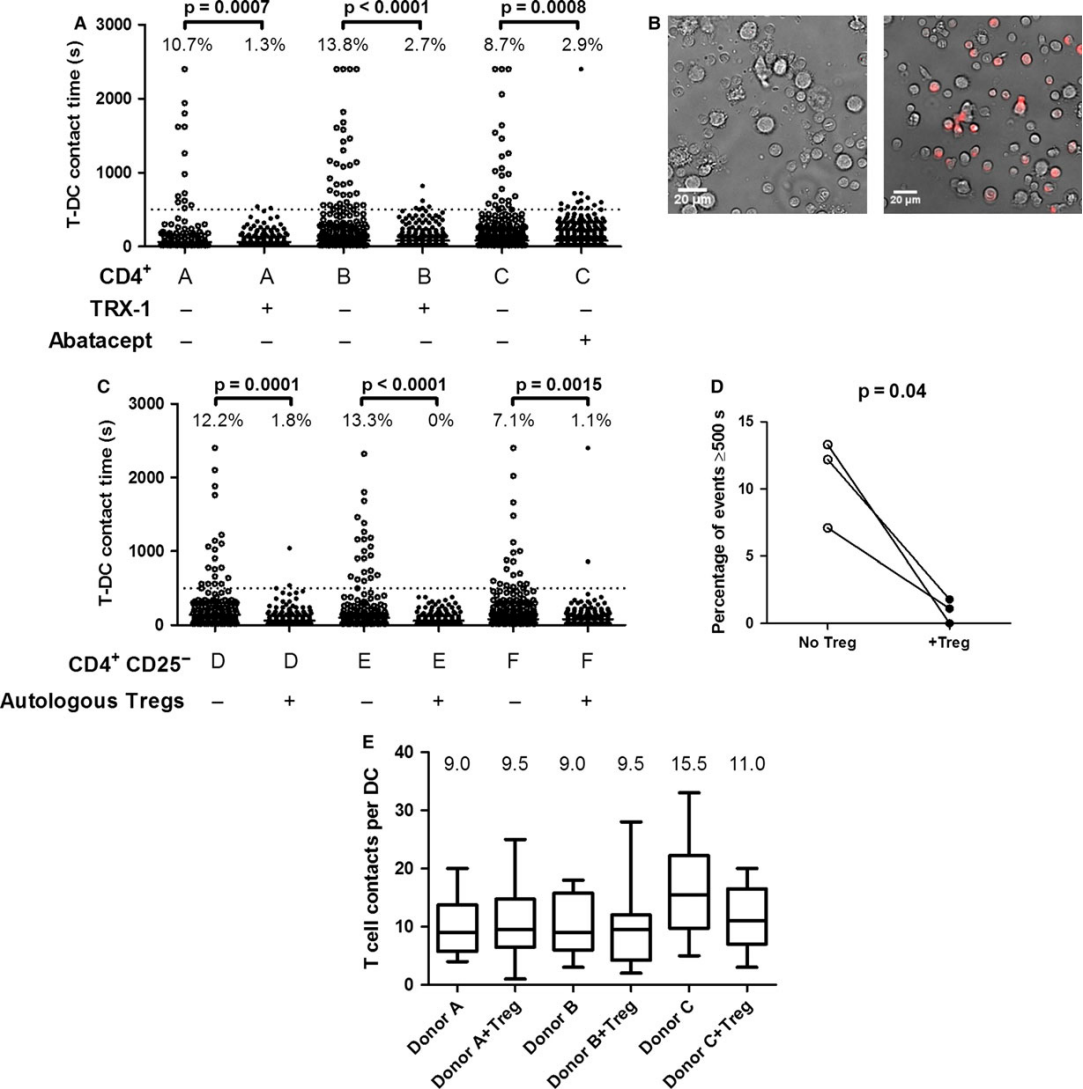
**Human T cells exhibit allogeneic T‐** **DC** **interactions that can be controlled by autologous Tregs.** (A) CD4^+^ T cells were sorted from buffy coats of three blood donors (A, B, and C) and allowed to interact with allogeneic monocyte‐derived DCs in the presence or absence of CD4 mAb TRX1 (1 μg/mL, donors A and B) or in the presence or absence of abatacept (1 μg/mL, donor C), as indicated below the graph. Dotted line indicates the 500‐s threshold above which contacts were nearly completely eliminated by TRX1 or abatacept. (B–D). CD4^+^ CD25^−^ T cells were sorted from buffy coats of three additional blood donors (D, E, and F) and allowed to interact with allogeneic monocyte‐derived DCs in the presence or absence of autologous activated and expanded CMPTX‐labeled CD4^+^ CD25^+^ CD127^lo^ Tregs. (B) Representative ×20 images show CD4^+^ CD25^−^ T cells (small) interacting with DCs (large) in the absence (left panel) or presence (right panel) of autologous Tregs (red). (C) Interaction times for each donor are shown in the presence or absence of autologous Tregs, as indicated below the graph. Percentage of events ≥500 s is shown above each column. For the comparison of interaction times in the presence or absence of Tregs with the Fisher exact test, p = 0.0001 (donor D), p < 0.0001 (donor E) and p = 0.0015 (donor F). (D) Pairwise comparison of the percentage of events ≥500 s in the presence or absence of Tregs for each individual. Paired t‐test, p = 0.04. (E) Box‐and‐whisker plots illustrating the number of CD4^+^ CD25^−^ T‐DC contacts per DC in each condition. Top and bottom horizontal bars indicate the maximum and minimum values. Top and bottom of each box represent the 25th and 75th percentiles. The horizontal bar in each box represents the median number of contacts. Numbers above each column are the medians. Data are derived from the two experiments depicted in panel C. DC, dendritic cell; T‐DC, T cell–dendritic cell; Treg, regulatory T cell.

We next sought to determine whether alloreactivity in polyclonal human CD4^+^ T cells could be quantified in a similar manner. Interactions of human CD4^+^ T cells from three healthy donors with allogeneic MoDCs were recorded using time‐lapse microscopy. Similar to our findings with mouse cells, the addition of a CD4 antibody, TRX1 42, that prevents human T cell activation (Figure 6A, second and fourth columns) or abatacept (Figure 6A, sixth column) indicated that a threshold of 500 s allowed discrimination between productive and nonproductive contacts.

Our laboratory has reported a method to expand highly potent human CD4^+^CD25^+^CD127^lo^ Tregs that can be cryopreserved and recovered without losing function 29, 43. Freshly thawed expanded human CD4^+^CD25^+^CD127^lo^ Tregs were labeled with the dye CMTPX. Sorted CD4^+^ CD25^−^ T cells were incubated with allogeneic MoDCs in the presence or absence of Teff–autologous CMTPX‐labeled Tregs (Figure 6B), and interaction times were determined. As shown in Figure 6C, CD4^+^ CD25^−^ T cells from three individual patients had >500 s T‐DC interaction frequencies of 12.2%, 13.3%, and 7.1%. Autologous expanded Tregs dramatically reduced these contacts, and in one case, they were eliminated completely (Figures 6C and D).

As in the experiments shown in Figure 5A, the total T cell number in the wells was kept constant to avoid crowding. Conventional T cells might thus have shown a reduction in prolonged interactions due to a diminished opportunity to interact with MoDCs; however, we observed either no such reduction or only a modest reduction in the number of T cell contacts with each DC (Figure 6E). Consequently, the profound inhibition of prolonged contact formation in the presence of Tregs was not due to a reduced T‐DC contact opportunity, confirming that alloreactive human T‐DC interactions can be modeled with this method. More important, the impact of expanded Tregs on T‐DC interactions *in vitro* reflects their ability to control human allograft rejection in functional *in vivo* models 29, 44, 45.

### Alloreactive immune synapse formation can be detected with imaging flow cytometry

Time‐lapse microscopy requires lengthy manual data analysis, an approach unlikely to be clinically applicable. Imaging flow cytometry has been used to examine immune synapses formed by monoclonal T cell populations 46, 47, 48, 49 or T cells driven by superantigens 50. We hypothesized that this technique could be used to enumerate alloreactivity in a polyclonal CD4^+^ T cell population. CBA CD4^+^ T cells were incubated with B6 DCs for 4 h; fixed and stained for T cell and DC markers, intracellular actin, and nuclear DNA; and then examined on an ImageStream IS100 imaging flow cytometer (Amnis Corp).

**Figure 7:**
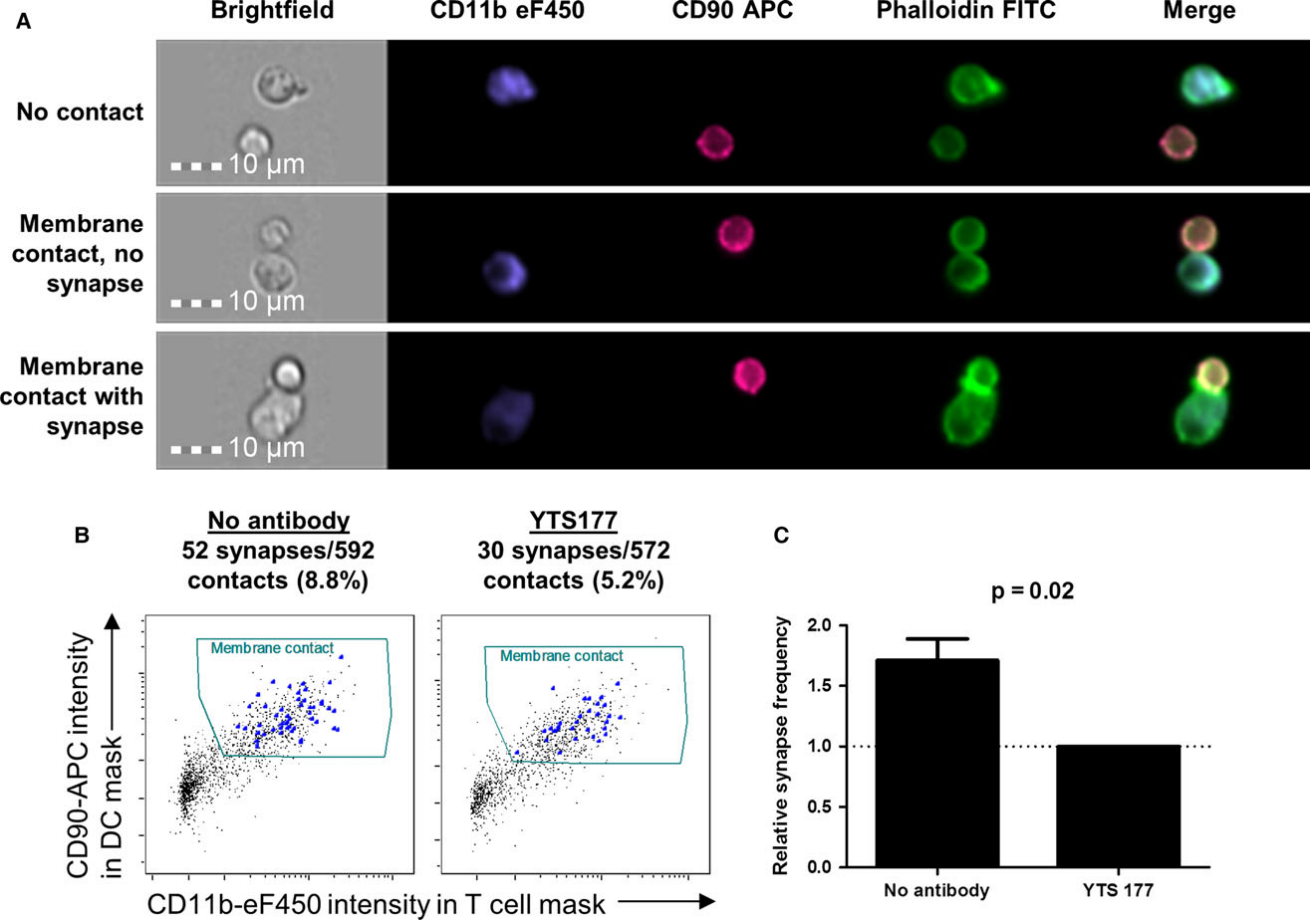
**Detection of synapses between** **CD** **4** ^**+**^ **T cells and allogeneic dendritic cells using imaging flow cytometry.** CBA CD4^+^ T cells were sorted and incubated at 37°C for 4 h with B6 DCs in the presence or absence of YTS177 (1 μg/mL). They were then fixed in the culture plate and stained for CD90, CD11b, actin (phalloidin‐FITC), and nuclei (7AAD) prior to acquisition on an ImageStream IS100 (Amnis Corp). (A) Examples of gated T cell–DC doublets are shown with no membrane contact (top panel), membrane contact without synapse formation (middle panel) and membrane contact with synapse formation (bottom panel), as indicated by the presence of actin polarization in the T cell. Nuclear staining is omitted for clarity. (B) Doublets with membrane contact were identified by plotting the presence of CD90 staining in the DC object mask against the presence of CD11b staining in the T cell object mask. Synapses were tagged within this gate by manually reviewing all images. Proportions of identified immune synapses within the membrane contact gate are shown above each plot. (C) In three independent experiments, there was a mean of 1.7‐fold more synapses in the absence of YTS177 than in its presence. Paired t‐test, p = 0.02. APC, allophycocyanin; DC, dendritic cell; FITC, fluorescein isothiocyanate.

Cell doublets containing one DC and one T cell were identified (the full gating strategy is shown in Figure S1). Doublets making an immune synapse could be distinguished from those making simple membrane contact based on phalloidin‐FITC staining that identified cytoskeleton rearrangements at the T‐DC interface (compare with Figure 7A center and lower panels). As in Figure 1, the addition of anti‐CD4 antibody (YTS 177) was used to determine whether imaging flow cytometry could distinguish between productive and nonproductive T‐DC contacts. YTS177 caused a ≈1.7‐fold reduction in the frequency of synapses in the appropriate contact gate (Figures 7B and C), providing a proof‐of‐concept demonstration that CD4^+^ T cell alloreactivity can be quantified in a semiautomated manner.

**Figure 8:**
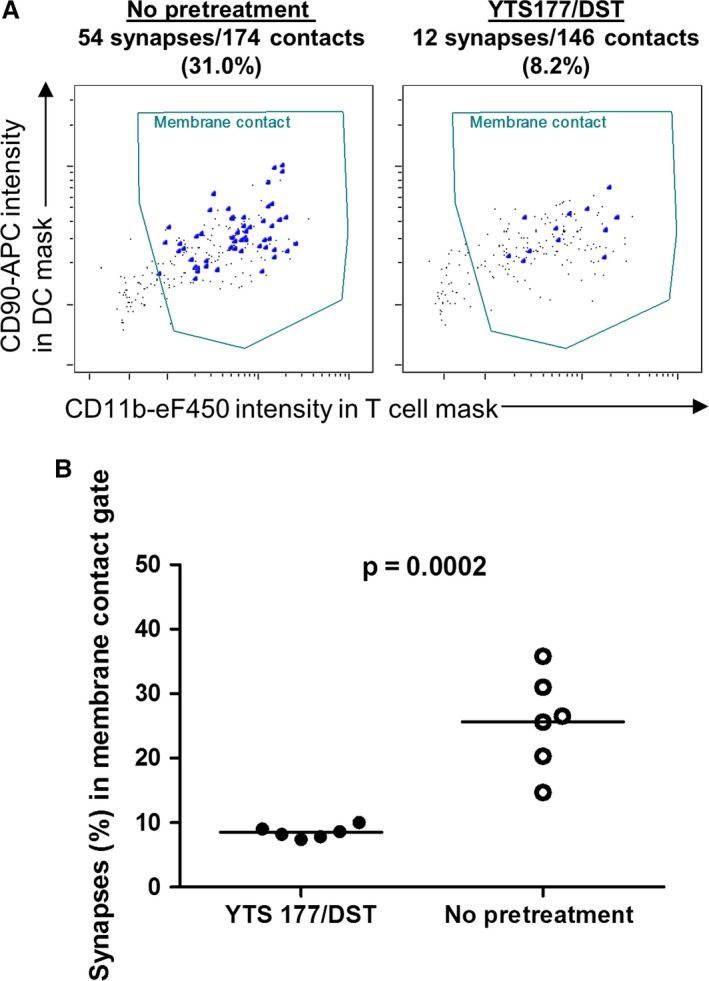
**Discrimination of tolerized and rejecting mice using imaging flow cytometry.** CBA mice were given YTS177/DST or no pretreatment followed by a B6 cardiac allograft, as in Figure 3. After 7 days, recipient splenic CD4^+^ T cells were cocultured with B6 DCs for 4 h and prepared for imaging flow cytometry, as described in Figure 7. (A) Representative plots of CD90 intensity in the DC object mask versus CD11b intensity in the T cell object mask are shown for a nonpretreated animal (left panel) and a YTS177/DST‐treated animal (right panel). Synapses (large dots) within the membrane contact gate were identified manually by an analyst blinded to treatment assignment. Synapse frequencies in the membrane contact gate are shown above each plot. (B) Synapse frequencies in the membrane contact gate for 6 animals per group in two independent experiments are shown. Unpaired t‐test, p = 0.0002. DC, dendritic cell; DST, donor‐specific transfusion.

Finally, to ascertain whether induction of allograft tolerance could be detected or predicted using imaging flow cytometry, CBA mice were left untreated or had tolerance induced using the 177/DST protocol before transplantation with B6 cardiac allografts. Seven days later, CD4^+^ splenocytes were isolated from individual mice; incubated with B6 DCs for 4 h; and then processed, stained and acquired on the IS100 imaging flow cytometer. The data were then analyzed in a blinded fashion, as described in Figures 7 and S1. Strikingly, the frequency of synapse formation was as high as 30% in nonpretreated animals, whereas YST177/DST‐treated animals exhibited a frequency of ≤10% (Figures 8A and B). These data are remarkably similar to those shown in Figure 3(B) and validate the imaging flow cytometry approach as a measure of CD4^+^ T cell alloreactivity.

## Discussion

T cells that subsequently become activated must arrest and remain in contact with APCs and form a mature immunological synapse 24, 26, 33, 35, 51, 52 characterized by actin polymerization and polarization 53. In this study, we assessed whether stable interactions between CD4^+^ T cells and allogeneic donor DCs *in vitro* could be used as a surrogate parameter for allograft rejection and unresponsiveness.

*In vitro* time‐lapse microscopy readily identified interactions between naive T cells and allogeneic DCs in both mouse and human. Anti‐CD4 antibody and CTLA4‐Ig, both known to block T cell activation 25, 37, 54, provided an unbiased threshold of 500 s for productive alloreactive T‐DC interactions. This is very similar to the figure of 400 s obtained by Sarris et al 25, who used MHC class II^−/−^ DCs to distinguish between immunologically relevant and irrelevant self‐restricted T cell responses. More recently, Dilek et al examined the effect of CD28 blockade on interaction times between a human CD4^+^ T cell clone and allogeneic B cells 55. CD28 blockade reduced T cell and B cell contact times to <300 s compared with >600 s in its absence 55. Our data are highly comparable with independent studies and provide a justification for measuring alloreactive T‐DC contact times *in vitro*.

Allograft rejection requires clonal expansion of alloreactive T cells driven by allogeneic APCs in graft‐draining secondary lymphoid organs 56. The data shown in Figure 2(C) demonstrate that this phenomenon can be detected *in vitro* by video microscopy and, importantly, that the technique can distinguish between responses in the draining and nondraining lymph nodes. Furthermore, the technique can also distinguish between mice rejecting an allograft and those in which tolerance was induced with a protocol that leads to long‐term graft survival (Figure 3) 20, 39.

Using two‐photon imaging of pancreatic lymph nodes, Tang and colleagues showed that Tregs reactive to an islet autoantigen reduced contact times between APCs and Teffs reactive to the same autoantigen 24. Similar findings were reported by Tadokoro et al in experimental autoimmune encephalomyelitis 26. In both cases, Tregs prevented Teff “swarming” and arrest on APCs. We have shown in this study that both mouse and human Tregs can reduce the frequency of stable T cell contacts with allogeneic DCs (Figures 5 and 6). Critically, when tested in the context of *in vivo* responses, this effect correlates with Treg function in a well‐characterized model of tolerance induction (Figures 4C–F). Consequently, a phenomenon previously shown to be relevant to Treg control of autoimmunity *in vivo* also occurs in an *in vitro* setting and reflects Treg‐mediated regulation of alloreactivity. Clinical trials of Treg cellular therapy are being conducted in hematopoietic stem cell transplantation 54, 57 and kidney transplantation 27, but methods for assessing their immunological impact have not been firmly established 58. Our data suggest a possible means of evaluating Treg efficacy.

CD4^+^ T cells from naïve mice (Figures 1D and F, 4B and D, and 5) and humans (Figures 6A and C) exhibited a frequency of prolonged T‐DC contacts of 5–15%. This is strikingly similar to the precursor frequency estimates, also 5–15%, of alloreactive T cells 59, 60, 61, 62. Significantly, Figure 3(B) shows that tolerance induction that leads to long‐term cardiac allograft survival 39 results in a reduction but not an abrogation of prolonged contacts. From a practical perspective, this emphasizes that if such an approach were to be considered for clinical evaluation, it would be essential to obtain baseline data before transplant to provide the reference for longitudinal follow‐up of rejection and quiescence. It should be noted that the data presented in this study were obtained using cryopreserved DCs (all results) and cryopreserved human T cells (Figure 6), so it is possible to envision conducting such assays in either deceased or living donor transplantation. Donor cells could be cryopreserved and then recovered at important junctures, such as during weaning of immunosuppression to assess antidonor T‐DC interactions.

We focused on CD4^+^ T cell responses principally because in the B6 to CBA mouse strain combination, long‐term graft survival can be achieved by manipulating only the CD4^+^ T cell compartment 39 and because graft survival in this model is dependent on CD4^+^ Tregs 20, 22, 63. These techniques, however, would lend themselves to an examination of alloreactive CD8^+^ T cell responses. Whether T cells interacting with autologous DCs via the indirect pathway could be detected using this method is unknown and was not tested in this study. The indirect pathway of alloantigen presentation is particularly important in the setting of chronic allograft dysfunction and is more relevant at later points after transplant 64, 65. Far fewer T cells are indirectly rather than directly alloreactive, and it seems unlikely that they could be quantified using time‐lapse microscopy. A high‐throughput strategy such as imaging flow cytometry might allow enumeration of these relatively rare events.

Enumeration of T cell–APC contacts by time‐lapse microscopy provides an important proof of concept but is very time consuming and could not be used clinically in its present form. Clinical translation will require semiautomated methods for detecting T‐DC contacts. The data shown in Figures 7 and 8 demonstrate that imaging flow cytometry could provide a high‐throughput alternative. This will require further evaluation and development, but our data support the concept that identifying a change in the frequency of prolonged T‐DC contacts or immune synapses could be useful in the management of transplant patients.

## Disclosure

The authors of this manuscript have no conflicts of interest to disclose as described by the *American Journal of Transplantation*.

## Supporting information


**Figure S1: Gating strategy used to identify doublets with membrane contact by imaging flow cytometry.** (A) Gates used to identify T cells in contact with DCs are shown. In‐focus events (top left) were plotted according to brightfield aspect ratio versus area to identify doublets. The latter were then plotted according to CD11b and CD90 staining. Double‐positive events were then further analyzed to include those containing only one DC and only one T cell (middle two plots). Finally, CD11b^+^ CD90^+^ doublets were further refined by including only those events with two 7AAD spots (i.e. nuclei) (bottom left histogram). Within this gate, T cells and DCs with physical membrane contact were identified by plotting CD90 staining intensity (T cell marker) in the DC object mask against CD11b staining intensity (DC marker) in the T cell object mask (bottom right plot). Synapses were identified within this gate by manual tagging of events with prominent polarization of actin staining within the T cell. (B) Images of a doublet are shown. The DC object mask is shown overlying the CD11b eF450 image, and the T cell object mask is shown overlying the CD90 APC image. Nuclear staining with 7AAD (yellow) is also shown. Merged image without masks is shown at right. The actin image (phalloidin–fluorescein isothiocyanate) is omitted for clarity. APC, allophycocyanin; DC, dendritic cell.

**Video S1: CBA Foxp3‐GFP CD4** ^**+**^ **T cells interacting with B6 CFP DCs.** CBA Foxp3‐GFP CD4^+^ T cells (2 × 10^5^) were combined with B6 CFP DCs (1 × 10^5^) in 200 μL media in a flat‐bottomed microscopy chamber at 37°C. Serial ×20 magnification images were acquired every 20 s for 40 min, generating 120 frames. Large blue DCs, colorless CD4+Foxp3^−^ effector T cells and green CD4^+^Foxp3^+^ regulatory T cells are visible. CFP, cyan fluorescent protein; DC, dendritic cell.

**Video S2: CBA Foxp3‐GFP CD4** ^**+**^ **T cells from rejecting mice interacting with donor‐specific B6 CFP DCs.** CBA Foxp3‐GFP mice were allowed to reject B6 cardiac allografts. At day 14 after transplant (all grafts rejected), their splenic CD4^+^ T cells (2 × 10^5^) were combined with B6 CFP DCs (1 × 10^5^) in 200 μL media in a flat‐bottomed microscopy chamber at 37°C and imaged, as described in the caption for Video S1. Large blue DCs, colorless CD4^+^Foxp3^−^ effector T cells and green CD4^+^Foxp3^+^ regulatory T cells are visible. CFP, cyan fluorescent protein; DC, dendritic cell.
