# Supplementary figures and images for: Quantification of CD4+ T Cell Alloreactivity and Its Control by Regulatory T Cells Using Time‐Lapse Microscopy and Immune Synapse Detection
Source: Am J Transplant. 2016 Jan 29;16(5):1394–407. doi: 10.1111/ajt.13607 (PMC4855688; doi:10.1111/ajt.13607)

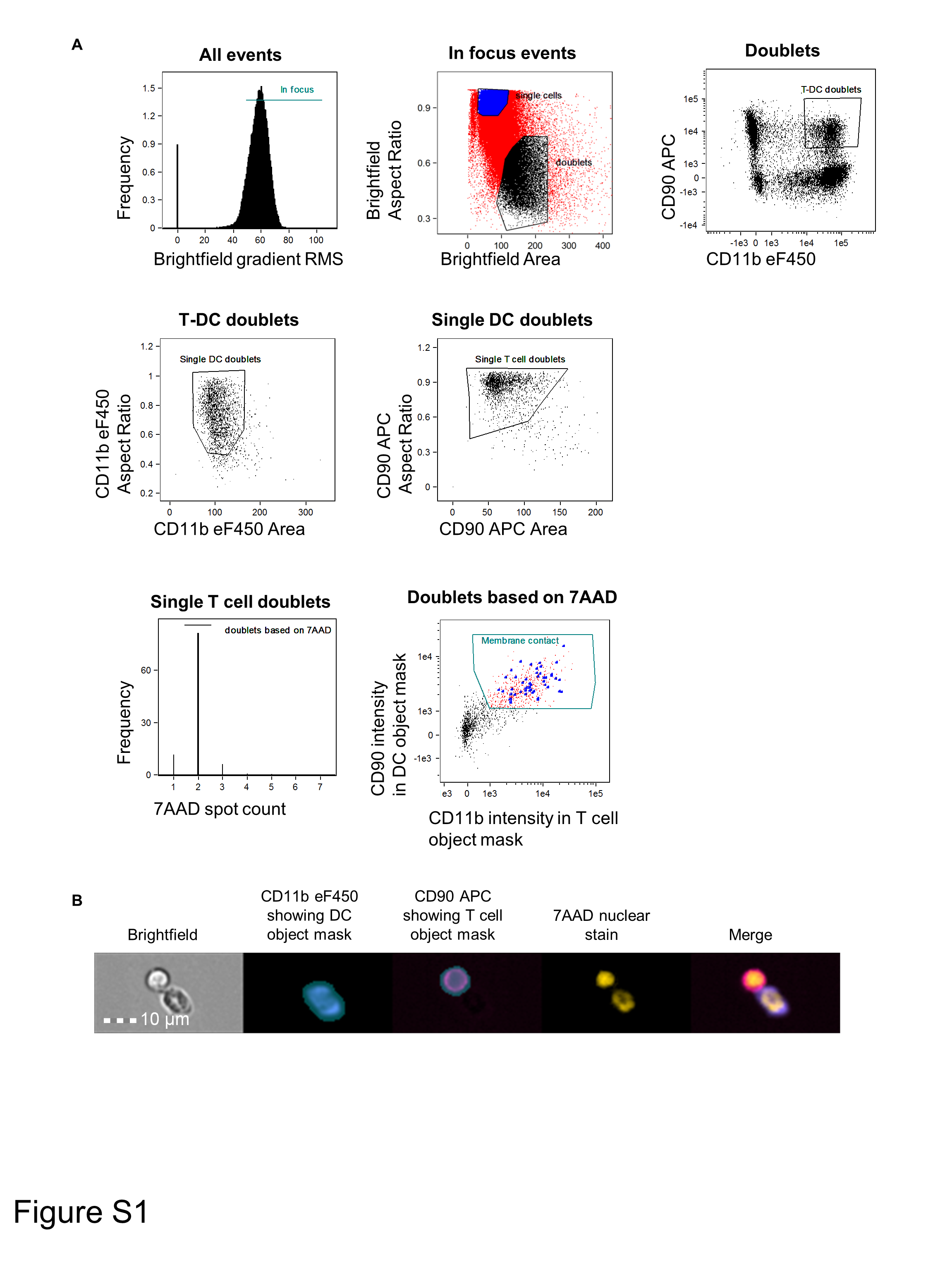

Supplement: Supplementary file 1 — Figure S1: Gating strategy used to identify doublets with membrane contact by imaging flow cytometry. (A) Gates used to identify T cells in contact with DCs are shown. In‐focus events (top left) were plotted according to brightfield aspect ratio versus area to identify doublets. The latter were then plotted according to CD11b and CD90 staining. Double‐positive events were then further analyzed to include those containing only one DC and only one T cell (middle two plots). Finally, CD11b+ CD90+ doublets were further refined by including only those events with two 7AAD spots (i.e. nuclei) (bottom left histogram). Within this gate, T cells and DCs with physical membrane contact were identified by plotting CD90 staining intensity (T cell marker) in the DC object mask against CD11b staining intensity (DC marker) in the T cell object mask (bottom right plot). Synapses were identified within this gate by manual tagging of events with prominent polarization of actin staining within the T cell. (B) Images of a doublet are shown. The DC object mask is shown overlying the CD11b eF450 image, and the T cell object mask is shown overlying the CD90 APC image. Nuclear staining with 7AAD (yellow) is also shown. Merged image without masks is shown at right. The actin image (phalloidin–fluorescein isothiocyanate) is omitted for clarity. APC, allophycocyanin; DC, dendritic cell. [file AJT-16-1394-s001.tif]
